# Enhanced Antimicrobial Activity of AamAP1-Lysine, a Novel Synthetic Peptide Analog Derived from the Scorpion Venom Peptide AamAP1

**DOI:** 10.3390/ph7050502

**Published:** 2014-04-25

**Authors:** Ammar Almaaytah, Shadi Tarazi, Ahmad Abu-Alhaijaa, Yara Altall, Nizar Alshar’i, Khaldon Bodoor, Qosay Al-Balas

**Affiliations:** 1 Department of Pharmaceutical Technology, Faculty of Pharmacy, Jordan University of Science and Technology, P.O. Box 3030, Irbid 22110, Jordan; E-Mail: yraltall@just.edu.jo; 2 Department of Applied Biological Sciences, Faculty of Science and Arts, Jordan University of Science and Technology, P.O. Box 3030, Irbid 22110, Jordan; E-Mails: shadytarazi89@hotmail.com (S.T.); akaboalhijaa11@sci.just.edu.jo (A.A.-A.); 3 Department of Medicinal Chemistry, Faculty of Pharmacy, Jordan University of Science and Technology, P.O. Box 3030, Irbid 22110, Jordan; E-Mails: nashari@just.edu.jo (N.A.); qabalas@just.edu.jo (Q.A.-B.); 4 Department of Biotechnology and Genetic Engineering, Faculty of Science and Arts, Jordan University of Science and Technology, P.O. Box 3030, Irbid 22110, Jordan; E-Mail: khaldon_bodoor@just.edu.jo

**Keywords:** antimicrobial peptides, peptide design, membrane-permeation, scorpion peptide, molecular modeling

## Abstract

There is great interest in the development of antimicrobial peptides as a potentially novel class of antimicrobial agents. Several structural determinants are responsible for the antimicrobial and cytolytic activity of antimicrobial peptides. In our study, a new synthetic peptide analog, AamAP1-Lysine from the naturally occurring scorpion venom antimicrobial peptide AamAP1, was designed by modifying the parent peptide in order to increase the positive charge and optimize other physico-chemical parameters involved in antimicrobial activity. AamAP1-Lysine displayed potent antibacterial activity against Gram-positive and Gram-negative bacteria. The minimum inhibitory concentration was in the range of 5 to 15 µM with a 10 fold increase in potency over the parent peptide. The hemolytic and antiproliferative activity of AamAP1-Lysine against eukaryotic mammalian cells was minimal at the concentration range needed to inhibit bacterial growth. The antibacterial mechanism analysis indicated that AamAP1-Lysine is probably inducing bacterial cell death through membrane damage and permeabilization determined by the release of β-galactosidase enzyme from peptide treated *E. coli* cells. DNA binding studies revealed that AamAP1-Lysine caused complete retardation of DNA migration and could display intracellular activities in addition to the membrane permeabilization mode of action reported earlier. In conclusion, AamAP1-Lysine could prove to be a potential candidate for antimicrobial drug development in future studies.

## 1. Introduction

Antimicrobial resistance currently represents one of the biggest challenges facing human health and the medical community. The misuse of antibiotics over the past decades has resulted in the emergence of several multi-drug resistant strains of microorganisms [1,2,3]. This problem has created an urgent need to develop novel classes of antimicrobial agents with different modes of action than the conventionally used antibiotics to control this problem [4,5]. Unfortunately, the number of antibiotics being developed and reaching the clinic in the recent years has declined significantly [6]. This decline is mainly attributed to the lack of investment by pharmaceutical companies in this field as their research attention has shifted in recent years towards more financially feasible and profitable areas of drug development [7]. One of the promising alternatives for conventional antibiotics is antimicrobial peptides (AMPs) as this group of molecules display potent activities against target cells, rapid killing kinetics and a broad spectrum of activity against different microbial strains [8,9,10,11]. Additionally, these peptides are usually gene coded in host organisms and are expressed either constitutively or induced by various external stimuli making the selection of resistant mutants to AMPs *in vitro* relatively difficult [10,11].

AMPs represent the first line of innate immune defense mechanism against infectious agents of a variety of organisms including insects, invertebrates, plants, birds and mammals [12,13]. Most AMPs share several common features, including a relatively small size (12–50 amino acid residues long), cationic nature (a net positive charge ranging from +2 to +9) and an amphipathic structure containing approximately 50% hydrophobic residues [14,15]. Among the various AMPs discovered, cationic α-helical peptides represent the most abundant and well studied group of AMPs [16]. Cationic α-helical AMPs are known to unlikely evoke bacterial resistance due to their ability to target bacterial membranes through electrostatic interactions and to bind lipid components of bacterial membranes. One of the major issues that have delayed the development of cationic AMPs is the ability of the peptides to cause significant damage to mammalian membranes and the lack of selectivity to distinguish microbial cells from mammalian cells on the basis of their different lipid membrane components. Thus, cationic AMPs displaying high selectivity towards microbial cells and low toxicity against mammalian cells and human erythrocytes would represent attractive candidates for use as therapeutic agents [17,18].

In pursuit of novel antimicrobial agents, several peptides from scorpion venoms with antimicrobial activity were identified recently [19,20,21,22]. The first scorpion AMP was Hadrurin from the venom of the scorpion *Hadrurus aztecus* [23], this discovery was followed by literature reports of several other scorpion AMPs. Recently, a novel AMP from the venom of the North African scorpion *Androctonus amoeruxi* was identified through molecular cloning and it displayed moderate broad spectrum antimicrobial activities against representative strains of Gram-positive, Gram-negative bacteria and yeast in the range of 20–150 μM [24]. Named as AamAP1, the peptide is composed of 17 amino acids and displays a cationic (+2 charge) α-helical structure. In addition to the moderate antimicrobial activity reported for AamAP1, the peptide also displayed significant hemolytic activity against sheep erythrocytes at concentrations that were employed in the antimicrobial studies and showed no selectivity against mammalian cells.

In the present study, we aimed to employ the antimicrobial peptide AamAP1 as a platform for the design of a novel peptide analog with enhanced antibacterial activity and decreased cytolytic activity against eukaryotic cells based on enhancing the net positive charge on the parent peptide while optimizing other physico-chemical properties of the peptide that are known to influence AMPs activity. The strategy applied here produced a novel peptide named AamAP1-Lysine with enhanced antimicrobial activity and decreased toxicity towards mammalian cells. AamAP1-Lysine could prove to be a potential candidate for antimicrobial drug development in future studies.

## 2. Experimental Section

### 2.1. Design of AamAP1-Lysine Based on Structural Determinants

AamAP1-Lysine was designed based on increasing the net positive charge of the parent peptide while relatively optimizing other structural determinants that control AMPs activity. These structural determinants include: hydrophobicity (H), hydrophobic moment (M_H_) and percentage helicity. Several peptide analogs were generated using this strategy and the peptide with the optimal structural determinants was selected (AamAP-1-Lysine) for further characterization of the effect of structural modification on the enhancement of peptide antimicrobial activity and target selectivity.

### 2.2. Molecular Modeling and In Silico Analysis of AamAP1-Lysine

The ProtParam software from the ExPASy server was employed for the evaluation of the physicochemical parameters of AamAP1-Lysine [25]. Structure prediction and the identification of the best template for homology modeling of AamAP1-Lysine were performed by the HHpred (HHsearch 2.0) software by HMM–HMM comparison [26]. The nearest template for reliable homology modeling was found with the RNA polymerase-associated protein Gp33 and showed a score of 20.9. Packing and solvent exposure characteristics were analyzed using the PROSA software [27]. Confirmation of the model reliability was performed using the I-TASSER software [28]. The RAMPAGE: Assessment of the Ramachandran Plot software was also used for the three dimensional structure validation [29]. The final model was visualized using Accelrys^®^ Discovery studio software.

### 2.3. Peptide Synthesis and Purification

AamAP1-Lysine was synthesized by the solid-phase method and Fmoc chemistry, purified by reverse phase high performance liquid chromatography using an acetonitirile/H_2_O-TFA gradient. The identity of AamAP1-Lysine was confirmed by ESI-MS mass spectrometry (GL Biochem Ltd., Shanghai, China).

### 2.4. Bacterial Strains

The bacterial strains used for the determination and testing of the antimicrobial activity of AamAP1 in this study were acquired from the American Type Culture Collection (ATCC, Manassas, VA, USA) and included: *Staphylococcus auerus* (ATCC 29213), *Staphylococcus auerus* (ATCC 43300), *Staphylococcus auerus* (ATCC 33591), *Staphylococcus epidermidis* (ATCC 12228), *Enterococcus faecalis* (ATCC 19433), *Escherichia coli* (ATCC 25922), *Salmonella enterica* (ATCC 10708), *Pseudomonas aerugino*sa (ATCC 9027), and *Klebsiella pneumoniae* (ATCC 13883).

### 2.5. Bacterial Susceptibility Assay

Susceptibility testing was performed by adopting the micro broth dilution method outlined by the Clinical and Laboratory Standards Institute (CLSI) guidelines using sterile 96-well plates [30,31]. Briefly, the organisms were removed from frozen glycerol stock and were grown overnight in Muller Hinton Broth (MH) and diluted to 10^6^ CFU/mL in culture medium prior to use. Fifty μL of twofold serial dilutions of AamAP1-Lysine in the concentration range of 1–20 μM were added to 50 μL bacteria in mid-log phase at a concentration of 10^5^ CFU/mL in 96 well microtitre plates. This was followed by incubating the plates for 18 h, at 37 °C, in a humidified atmosphere. Inhibition of microbial growth was determined by measuring the absorbance at *λ* = 570 nm with a microplate reader. Antibacterial activity was expressed as the minimal inhibitory concentration (MIC), for the minimum bactericidal concentration (MBC), 10 μL of the well contents were spread on agar and grown at 37 °C for 24 h or 48 h. The MBC was determined as the lowest concentration that resulted in <0.1% survival of the subculture. All MIC and MBC determinations were made in triplicate.

### 2.6. β-Galactosidase Assay

The ability of the AamAP1-Lysine to inflict damage on the cytoplasmic membrane of bacterial cells and cause significant membrane permeabilization can be assessed by measuring the release of cytoplasmic β-galactosidase from *E. coli* (ML-35) cells into the culture medium using ONPG as substrate and as described previously [32]. *E. coli* cells were grown in LB medium/5% lactose at 37 °C to an OD_590 nm_ of approximately 0.8. The cells were later washed twice and centrifuged for 10 min at 1.4 × *g* followed by resuspending the cells in PBS at the same OD. About 1 × 10^6^ cells reconstituted in 100 μL PBS and were incubated with different concentrations of AamAP1-Lysine. The hydrolysis of ONPG to O-nitro phenol by β-galactosidase enzyme was monitored at 405 nm using an ELISA plate reader over different time points (10, 20, 30, 40, 50, 60 and 120 min).

### 2.7. Extraction of Genomic DNA

The Wizard^®^ Genomic DNA Purification Kit (Promega, Madison, WI, USA) was used for the isolation of *E. coli* bacterial genomic DNA according to the manufacturer instructions. The purity of the extracted DNA was considered of good quality if an optical ratio of DNA was in the range of OD260/OD280 ≥1.8. A Thermo Scientific Nanodrop 1000 instrument (Wilmington, DE, USA) was used for determining the concentration of purified *E. coli* DNA at an absorbance of 260 nm and 280 nm.

### 2.8. DNA Gel Retardation

The effect of AamAP1-Lysine on DNA gel retardation was preformed as described previously with minor modifications [33]. Briefly, increasing amounts of AamAP1-Lysine in 30 μL binding buffer (10 mM Tris–HCl, 1 mM EDTA buffer, pH 8.0) was mixed with 500 ng of the genomic DNA. DNA migration was assessed by gel electrophoresis using a 0.8% agarose gel. The bands detected by fluorescence of eithidium bromide (EB). Finally gel retardation was visualized under UV illumination using a GelDoc-It® imaging system (UVP, Upland, CA, USA).

### 2.9. Erythrocyte Hemolysis Assay

The determination of the hemolytic activity of AamAP1-Lysine was performed as reported previously [34]. Briefly, a 4% suspension of human erythrocytes was incubated with the peptide for 60 min. Centrifugation with 0.9% NaCl was used for washing the human erythrocytes several times followed by incubation for 60 min, at 37 °C. The cells were then centrifuged at 900× *g* for 5 min until the supernatant was separated from the pellet. Two hundred µL were transferred from each sample supernatant as 4 replicates into a 96-well plate and their absorbance measured at 570 nm. 0.9% NaCl was used as the negative control, 0.1% Triton X-100 as the positive control. The percent hemolysis was calculated using the following equation: hemolysis = (A − A0)/(AX − A0) × 100, where A is OD 570 nm with the peptide solution, A0 is OD 570 nm in NaCl, and AX is OD 570 nm with 0.1% Triton X-100.

### 2.10. Cell Culture

The following cell lines (HEK 293 Human Embryonic Kidney 293 cell line and Vero African green monkey kidney epithelial cell line) , purchased from the ATCC were routinely cultured in RPMI 1640 or Dulbecco’s modified Eagle’s medium (DMEM) containing 10% fetal bovine serum (FBS), 1% l-glutamine, 1% sodium pyruvate, 50 U/mL penicillin and 50 mg/mL streptomycin. The cells were seeded into 150 cm^2^ culture flasks. Cells were cultured as monolayers in a humidified environment of 5% CO_2_ 95% air at 37 °C.

### 2.11. Cell Proliferation Assay

The cytotoxicity of AamAP1-Lysine against cultured cells was determined by the MTT assay. Each cell line used in this experiment was seeded at a density of 5 × 10^3^ cells per well into a 96-well microtitre plate containing 200 µL serum free media. Cells were incubated with various concentrations of the peptide. After 24 h of incubation, 20 μL of 5 mg/mL of 3-(4,5-dimethylthiazol-2-yl)-2,5-diphenyltetrazolium bromide (MTT) was added and incubated for 4 h. Conversion of MTT into purple formazan by metabolically active cells indicates the extent of cell viability. The medium was replaced by 200 µL of dimethylsulfoxide (DMSO) and mixed to dissolve the formazan crystals that had developed. Absorbance was measured using an ELISA Microplate Reader at 550 nm. The GraphPad Prism software was used for statistical analyses. 

## 3. Results

### 3.1. AamAP1-Lysine Design and Structural Modeling

**Figure 1:**
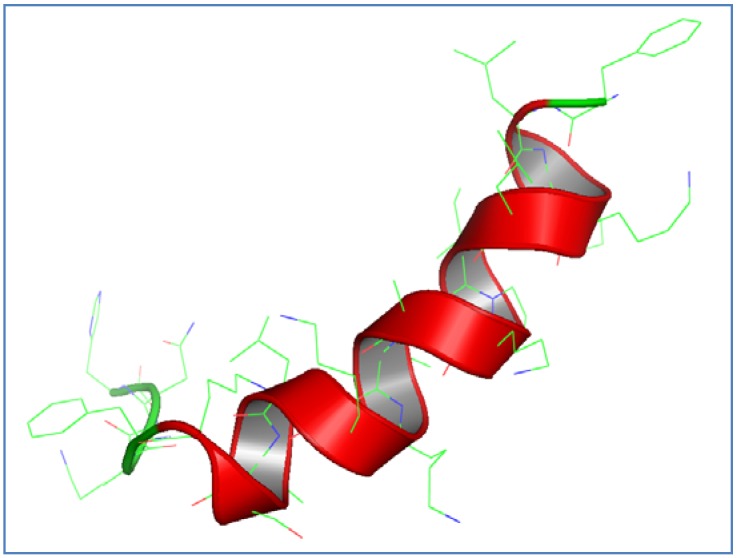
Three-dimensional structural modeling of AamAp1-Lysine. Red regions correspond to helical structures within the peptide while the green regions represent hinged regions and unordered conformations, respectively. The structure was visualized using Accelrys Discovery studio software.

The peptide sequence of the natural scorpion venom antimicrobial peptide AamAP1 was used as a platform for the development of a novel synthetic peptide analog with enhanced antimicrobial activity. AamAP1 displays a net positive charge of +2, percentage helicity of 66.6%, a hydrophobic moment of 0.44 and a hydrophobicity average of 0.90 (Table 1). The rationale used for designing the peptide analog was to increase the positive charge on the parent peptide by substituting several amino acids found on the primary sequence with lysine amino acids while relatively optimizing other physico-chemical parameters of the parent peptide that are known to influence its activity such as hydrophobicity, hydrophobic moment, and helicity. As charge is considered a crucial structural determinant of antimicrobial activity as it is believed to be responsible for the initial interaction of cationic AMPs with the negatively charged bacterial membranes, an increase in the net positive charge is expected to enhance the antimicrobial activity of the parent peptide and minimize its toxicity. As shown in Table 1, five lysine amino acids were employed for substitution at positions 4, 8, 11, 12 and 16 on the parent peptide. The resultant peptide analog (AamAP1-Lysine) displays a net positive charge of +6, percentage helicity of 88.3%, a hydrophobic moment of 0.61 and a hydrophobicity average of 0.61. The N-terminal amidation was unmodified due to several studies reporting loss of activity upon loss of amidation and the role on the N-terminal amidation in enhancing the biological activity of AMPs. Theoretical analysis of AamAP1-Lysine displayed that the peptide is adopting an alpha-helical structure when generated by the different methodologies mentioned earlier. The three dimensional model of AamAP1-Lysine revealed the peptide to exhibit an alpha helix conformation as generated by two different methodologies described previously (Figure 1). The Ramachandran plot was used for model validation and confirmed that 100% of the amino acids participating in forming the secondary structure of the peptide are in favored regions in relation to the phi and psi torsion angles of generated model. Additionally, a generated z-score of −1.78 was reported for AamAP1-Lysine using the PROSA II software which indicates that the generated model is of good quality.

**Table 1:** The amino acid sequences and properties of the peptides employed in this study.

Peptide	Sequence	Hydrophobicity (H)	Hydrophobic moment (M_H_)	Helicity
AamAP1	FLFSLIPHAIGGLISAFK	0.9	0.44	66.60%
AamAP1-Lysine	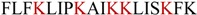	0.61	0.61	88.3%

### 3.2. Antimicrobial Activity of AamAP1-Lysine

The broth micro-dilution method was employed for the investigation of the antimicrobial activity of AamAP1-Lysine against five strains of Gram-positive bacteria and four strains of Gram-negative bacteria. The MIC values of AamAP1-Lysine against the bacterial strains are summarized in Table 2. The modified antimicrobial peptide analog AamAP1-Lysine displayed potent antimicrobial activities against all bacterial strains studied with *S. epidermidis*, *S. aureus* (29213), *S. aureus* (43300), *S. aureus* (33591), *E. faecalis*, *P. aeruginosa* and *K. pneumoniae* being the most sensitive with MIC value of 5 µM. Additionally, the MIC value reported for AamAP1-Lysine against both Gram-negative strains of *E. coli* and *S. enterica* was 7.5 µM. The MBC values reported for AamAP1-Lysine against all bacterial strains studied were equal to the MIC values which indicate that the peptide analog is exhibiting a bactericidal antimicrobial nature.

**Table 2:** Minimum inhibitory concentrations (MICs) of AamAP1-Lysine against the test microorganisms employed in this study.

Strain (Gram positive)	ATCC	MIC (µM)
*Staphylococcus epidermidis*	12228	5
*Staphylococcus aureus*	29213	5
*Staphylococcus aureus*	43300	5
*Staphylococcus aureus*	33591	5
*Enterococcus faecalis*	19433	5
**Strain(Gram negative)**	**ATCC**	
*Eshereschia coli*	25922	7.5
*Salmonella enterica*	10708	7.5
*Pseudomonas aerugino*sa	9027	5
*Klebsiella pneumoniae*	13883	5

### 3.3. Hemolytic Activity of AamAP1-Lysine against Human Erythrocytes

The hemolytic activity and potential toxicity of AamAP1-Lysine against human erythrocytes was examined at a concentration range of 1 µM to 100 µM. Results of the hemolytic activity of AamAP1-Lysine are presented in Table 3. At concentrations of 5 and 10 µM, which corresponds to the MIC & MBC concentration values reported for AamAP1-Lysine, the peptide induced minimal hemolysis of (0%–1.38%) when incubated with human RBCs for sixty minutes. At a concentration of 80 µM, that is ten times greater than the geometric MIC value reported for the peptide against all tested bacterial strains, AamP1-Lysine induced a percentage hemolysis of 29.3%. These results clearly indicate that AamP1-Lysine exhibits significant antimicrobial specificity and cell selectivity and weak hemolytic and RBC cytotoxicity.

**Table 3:** Hemolytic effect of AamAP1-Lysine on human erythrocytes after 60 min of incubation.

Peptide concentration (µM)	Hemolysis (%)
1	0
5	0
10	1.38
20	7.25
40	16.58
60	21.29
80	29.32
100	38.25

### 3.4. Viability of Eukaryotic Cells in Culture

**Figure 2:**
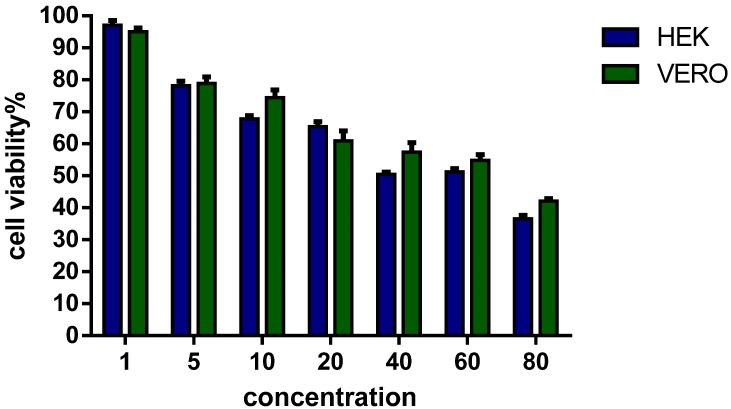
Cell survival curves as measured by MTT assay for AamAP1-Lysine against two different eukaryotic cell lines HEK 293 and Vero cells lines. Cells were incubated with various concentrations of the peptide, for 24 h, at 37 °C. Control cells represent 100% proliferation, and the mean absorbance of treated cells was related to control values to determine sensitivity. Error bars represent standard error from mean cell proliferation as determined by repeated experiments.

In addition to the hemolytic and cytolytic effects conducted with human erythrocytes, the antiproliferative activity of AamAP1-Lysine on the viability of two different eukaryotic cell lines (HEK 293 and Vero) was investigated and performed using the MTT assay. As shown in Figure 2, in the concentration range of AamAP1-Lysine needed to inhibit microbial growth (MIC of 5–10 µM) against all bacterial strains employed in this study, the peptide exerted moderate cytotoxicity and the percentage cell viability was reported to be of 73% on average against both eukaryotic cell lines tested. At higher concentrations of 60–80 µM, a significant increase in cytotoxicity is observed with percentage cell viability varying from 55% to 42%. These results suggest that application of AamAP1-Lysine at MIC concentrations can cause mild disruption of eukaryotic cells.

### 3.5. Cytoplasmic Membrane Permeability

**Figure 3:**
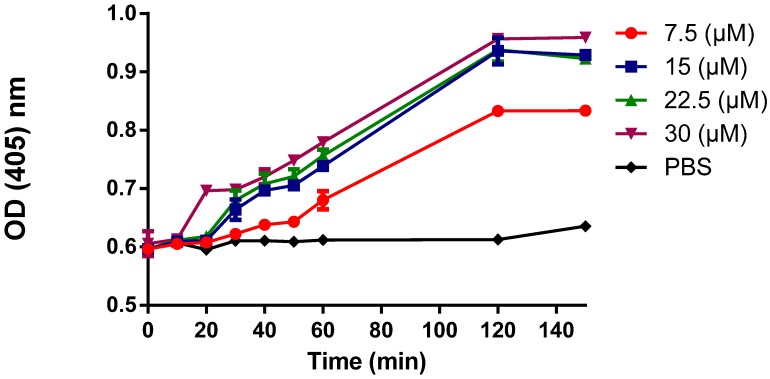
Release of cytoplasmic β-galactosidase from *E. coli* bacterial cells after treatment with four concentrations of AamAP1-Lysine (7.5 µM, 15 µM, 22.5 µM and 30 µM) or PBS (Negative control). The Y axis represents the optical density (OD) at 405 nm. Data are representative of two independent experiments.

To assess the effect of AamAP1-Lysine on the integrity of the cytoplasmic membrane of bacterial cells, the intracellular leakage and release of β-galactosidase from damaged bacterial cells degrades OPNG producing *o*-nitrophenol which can be monitored spectrophotometrically at 405 nm. As shown in Figure 3, after treatment with different concentrations of AamAP1-Lysine, β-galactosidase was released from *E. coli* cells with a lag time of about 15 min and caused a steady increase in OD values over time which corresponds with *o*-nitrophenol formation. Results suggested that AamAP1-Lysine could permeabilize cytoplasmic membrane of *E. coli* cells in a dose dependent manner.

### 3.6. DNA-Binding Activity

**Figure 4:**
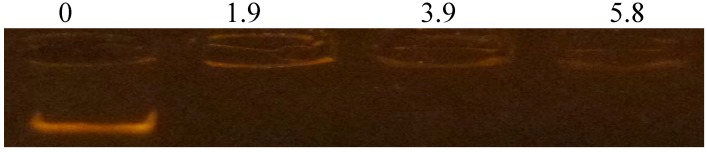
Gel retardation assays. Binding was assayed by the inhibitory effect of AamAP1-Lysine on the migration of DNA bands. Various amounts of peptides were incubated with 500 ng of *E. coli* genomic DNA at room temperature for 10 min, and the reaction mixtures were applied to a 0.8% agarose gel. The gel was visualized after ethidium bromide staining and UV irradiation. The numbers above the lanes represent the weight ratio (peptide/DNA).

The ability of AamAP1-Lysine to bind DNA and retard its migration was evaluated using an electrophoretic gel mobility shift assay. Different ratios of peptide to DNA were mixed with a fixed amount of *E. coli* genomic DNA, after which the mixture was electrophoresed on agarose gel. As shown in Figure 4. AamAP1-Lysine could interact with bacterial genomic DNA and cause a significant retardation in its movement starting from a peptide/DNA ratio of 1.9 which corresponds to 0.216 µg of AamAP1-Lysine. At higher concentrations, the peptide also caused complete retardation of DNA migration indicating the peptide could possess intrinsic DNA binding abilities.

## 4. Discussion

Scorpion venoms contain a cocktail of bioactive peptides that display numerous biological activities [35]. Several scorpion venom peptides have been reported to display antimicrobial activities [35], and more than 40 scorpion venom antimicrobial peptides have been indentified and functionally characterized in the last decade [36]. The majority of these scorpion antimicrobial peptides display potent broad spectrum antimicrobial activity against representative strains of planktonic and multidrug resistant bacteria [36]. One of the major drawbacks of scorpion AMPs is the lack of evident microbial cell selectivity against target cells as the peptides induce strong hemolytic effects against mammalian cells and human erythrocytes [36]. The majority of scorpion AMPs are composed of 13–55 amino acids and display a cationic amphipathic alpha-helical structure and fit into the family of host defense peptides or AMPs in terms of activity and the characteristic structural determinants responsible for the mechanism of action and the molecular interaction between the peptide and target cellular membranes. AMPs have generated a great interest for their potential as drug candidates for drug development due to their potent antimicrobial activity and different mechanism of action when compared with conventional antibiotics. Several studies and an extensive amount of research have been conducted to elucidate the mechanism of action of AMPs and several theories have been provided including: the barrel stave model, the torroidal pore model and the carpet mechanism [37]. However, the exact mechanism of action of AMPs remains unknown and controversial till now. It is generally accepted that AMPs kill bacterial cells by mainly targeting bacterial membranes and inhibiting bacterial reproduction by binding microbial intracellular organelles [38,39]. Additionally, the activity of AMPs and cell selectively has been attributed to several structural determinants within the primary sequence of the peptide such as charge, helicity, hydrophobicity and hydrophobic moment [40]. Recently, a novel scorpion AMP has been identified through molecular cloning from the venom derived cDNA library of the North African scorpion *Androctonus amoeruxi*, named as AamAP1, the peptide displayed moderate antimicrobial activity in the range of 20–150 µM against representative strains of Gram-positive and Gram-negative bacteria and suffered from significant toxicity against human erythrocytes [36]. In this project AamAP1 was used as a platform for the development of a new synthetic peptide analog with enhanced antimicrobial activity and decreased toxicity against mammalian cells. The synthetic analog, was modified to display an increase of +4 in the net positive charge while optimizing other structural determinants involved in the antimicrobial activity. Four amino acids on the parent peptide were substituted with lysine residues in order to increase the overall charge of the peptide. The resultant peptide analog, named as AamP1-Lysine displayed a net positive charge of +6 due to lysine substitution and exhibited a decrease in hydrophobicity from 0.90 to 0.61 and an increase in and hydrophobic moment from 0.44 to 0.61 respectively. Moreover, the amino acids substitution lead to an increase in percentage helicity from 66.3% to 83.3% as predicted by the NPS secondary structure prediction analyses (Swiss Model Workspace). The resultant changes in the structural parameters of AamAP1-Lysine lead to significant enhancement in the antimicrobial activity and cell selectivity of the peptide towards microbial cells. AamAP1-Lysine managed to inhibit bacterial growth for both Gram-positive and Gram-negative bacteria with an average geometric MIC of 7.5 µM compared with the parent peptide which displayed an average MIC geometric of 85 µM. This 10-fold increase in potency and antimicrobial activity could be explained by the optimized structural parameters AamAP1-Lysine possesses when compared to the parent peptide. Charge is considered crucial to the antimicrobial activity as it is responsible for the initial interaction of the antimicrobial peptides with the negatively charged phospholipids on microbial membranes [41]. Bacterial membranes are rich in the acidic phospholipids PG, PS, and CL which contributes collectively in conferring the overall negative charge on bacterial membranes [42]. Moreover, LPS and teichoic or teichuronic acids of Gram-negative and Gram-positive bacteria, impart additional negative charge to the surfaces of these respective organisms [42]. Thus, an increase in the cationic charge of AMPs could increase the electrostatic binding of the peptide to its target cell and consequently facilitate the killing mechanism. The increase in antimicrobial activity was also accompanied by a decrease in the hemolytic activity of the peptide against human erythrocytes. As reported earlier AamAP1-Lysine caused negligible hemolysis at MIC concentrations when compared with the parent peptide which was found to be strongly hemolytic and displayed weak selectivity towards microbial cells. The cytotoxicity of the peptide studies were also confirmed using the MTT cytotoxicity assay against human HEK 293 and Vero cell lines as the peptide caused mild disruption of eukaryotic cells. This improvement in the toxicity profile and selectivity index could also be attributed to the increase in cationic charge and improved binding to microbial membranes. Other factors could have also contributed to the improvement of the selectivity index as the helicity of the peptide and hydrophobicity that were increased due to structural modifications on the parent peptide and are considered as major contributors of effective antimicrobial activity and target selectivity [43]. To confirm the helicity and the adoption of an α-helical structure by AamAp1-Lysine, the secondary structure of the peptide was constructed using *in silico* homology modeling and the model generated clearly displays a stable alpha helical structure in confirmation with our preliminary analysis results.

Finally and in order to confirm the membrane targeted mechanism of action of AamAP1-Lysine, the membrane permeabilization ability of the peptide in addition to its ability to bind genomic bacterial DNA was investigated. AamP1-Lysine managed to increase *o*-nitrophenol formation as indicated by the increase in disruption of the membrane’s integrity due to leakage of intracellular β-galactosidase in a dose dependent manner. Moreover the peptide was able to cause complete retardation of the migration of bacterial genomic DNA at all peptide concentrations employed. This could indicate that the peptide could display dual mechanisms by killing bacteria due to membrane damage in addition to the binding of genomic DNA.

## 5. Conclusions

In summary, we report the design and functional characterization of the antimicrobial and cytotoxic activity of a synthetic scorpion venom peptide analog with enhanced potent antibacterial activities and low hemolytic and cytotoxic activities against eukaryotic cells. Several additional studies are needed to assess the activity and potential toxicity of AamAP1-Lysine using *in vivo* animal models but the preliminary results obtained from this study indicate that AamAP1-Lysine could have the potential for the development into an effective novel antimicrobial agent.
